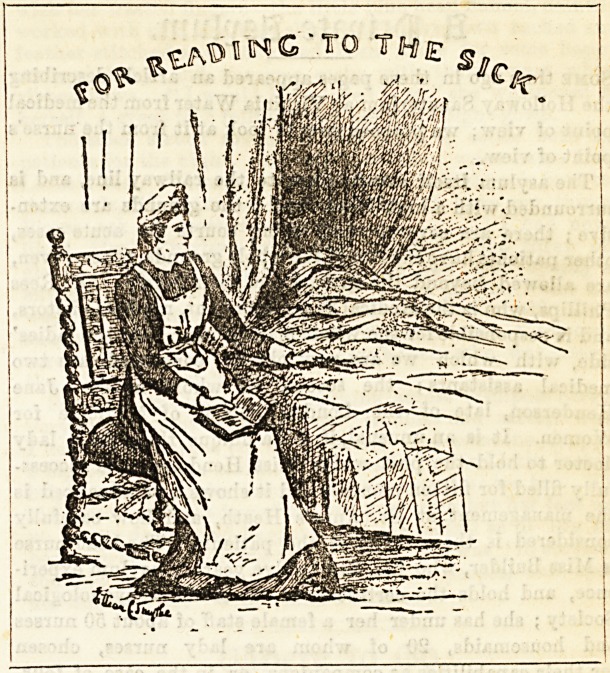# The Hospital Nursing Supplement

**Published:** 1892-04-02

**Authors:** 


### "The Hospital" Nursing Mirror.

Being the Extra Nursing Supplement of "The Hospital" Newspaper.

Contributions for this Supplement should be addressed to the Editor, The Hospital, 140, Strand, London, W.C., and should have the word "Nursing" plainly written in left-hand top corner of the envelope.

### En Passant.

THE REGISTRATION OF NURSES.—We are requested to state for the information of Lady Superintendents, Matrons, and their assistants who are opposed to the proposals of the British Nurses' Association, that they can procure for signature copies of the petition of Hospitals and Nurse-Training Schools, and of others directly interested in the advancement of trained nursing, on written application to Mr. I. G. Wainwright, Treasurer, St. Thomas's Hospital, Albert Embankment, London, S.E. Early application should be made by those who desire to attach their signatures to this petition to the Queen's Most Excellent Majesty in Council.

BURTON INSTITUTION. — Miss Carson and her district nurses paid 15,730 visits last year. There are two nurses at work for night cases only, and a third is shortly to be added to the staff. The ten private nurses attended 102 cases; and the debt on this branch has been reduced to £15.

THE PRIVATE NURSE.—Nurses are only human, and it is only natural that there should be in their ranks some black sheep; unfortunately these women soon get dismissed from institutions and hospitals, and then they prey on the public. Thus it comes that we hear of private nurses who drink, who get into debt, who misbehave in various ways. It should be the object and aim of every nurse to prevent these black sheep from procuring a certificate, or gaining the title of nurse. Such women dare not refer to any known institution or hospital, but there is danger ahead of every woman who gains her living by nursing being put on the same level. Should this be?

NURSES' BENEFICIAL ASSOCIATION.—It was only two years ago we noted the start of this society in Philadelphia, and already our go-ahead American sisters can tell of its tried success. The Association is a sort of small local pension fund, providing for members in case of illness and death; it has an endowed bed in the hospital, and under its auspices lectures have been given. The following figures are taken from the report: Number of members added, 57; number receiving benefits, 22; number of members occupying free room, 6; number of days occupied, 142. Evidently American nurses are also excellent in combining; it must be the discipline of hospital life, we think, which so enlarges the mind, that nurses, above other women, can co-operate together without quarrelling over trifles or fretting over the occasional difficulties which must arise in every organisation.

SUNDERLAND INSTITUTE.—The annual meeting was held on March 11th, the Mayor in the chair. Dr. Maling, the Hon. Secretary, submitted the third annual report, in which pleasure was expressed at the satisfactory way the work had gone on during the past year. The good work done by the institute was being more and more appreciated by the public. The institute had been able to pay its way during the year by the aid of donations and increased fees. On December 31st there were 17 nurses in the institute, against 12 at the beginning of the year, and the demand for their services had been well sustained. The earnings of the nurses during the year amounted to £492 9s. 6d., as against £477 5s. 6d. during the previous year. The number of cases attended by the private nurses was 116, as against 92 in 1890. The fees for patients nursed in the institute during 1891 amounted to £50 8s., as against £23 17s. of 1890. The district nurse had attended 120 patients, and had paid 2,002 visits, as against 82 and 1,809 in the previous year. In the course of her visits she had come across much poverty and misery. Her work had been much appreciated and the applicants for her services were, in many cases, friends of th patients.

LITTLE CHILDREN.—The annual meeting took place at the Home and Hospital for Incurable Children, Maida Vale, on Saturday afternoon. Several pleasant speeches were made, and Canon Duckworth was a most genial chairman. The cordial good feeling towards the work and workers which pervaded the meeting formed a distinct characteristic of the day, and the appreciative references to the Matron's influence were loudly applauded by all the visitors. The courteous welcome given at all times to all comers, being no less noteworthy than the good work so faithfully performed by Matron and nurses in an institution which needs to be doubled or trebled in size to meet the increasing demands for admission.

ST. MARY'S HOME, SOUTHAMPTON.—The sixteenth year of this institution has been marked by the building of a model home by Mr. Garton. The balance-sheet shows a pleasant balance. The work of nursing the sick has largely increased, partly from the nurse being each year better known and valued, and partly from the epidemic of influenza, but perhaps next to the witness borne by the doctors as to the great efficiency of Nurse Allen, the best testimony is the very sincere pleasure the new Home has given to those who have benefited by her skill and ready sympathy, and the often outspoken comments "that whatever contributed to the health and comfort of the nurse was well deserved." As this scheme began when there was much prejudice against nursing, it is encouraging to chronicle its success.

BICESTER NURSING HOME.—The annual meeting was held on March 16th. The Rev. J. C. Blomfield (Secretary) gave his report, which was as follows: We have now arrived at the close of a sixth year in the history of the nursing scheme. It has been, especially towards the end of it, a year distinguished from many others by the prevalence of unusual sickness and mortality. The work done in the course of the past year has been of the ordinary kind, the nurses having had under their care 82 cases of sickness among the poor of the town, to whom they paid 3,382 visits, 15 cases of private patients, and 13 patients in the Home. The question now coming into prominence is that of a more permanent and certain support of this institution. Six years ago it was a tentative scheme, of modest proportions, with receipts amounting to £94 1s. 10d. to meet an expenditure of £89 5s. 1d. yearly. At the present time both receipts and expenses have largely increased.

BIRMINGHAM INSTITUTION.—The annual meeting of the Midland Nurses' Training Institution was held on March 18th, the Mayor presiding. The report stated that the number of nurses on the staff was 96, of whom 82 were trained, and 14 probationers. The number of families attended during the year was 780. Infectious cases: 33 scarlet fever, 16 typhoid, and 18 diphtheria; and 84 families had been nursed through influenza, in some cases several members of the household being ill from this scourge at the same time. Eleven probationers had completed their training either at the General Hospital, Birmingham, or the General Hospital, Wolverhampton, or the General Infirmary, Worcester; while two who had received some useful special training in the Royal Orthopædic Hospital, would complete their term in some general hospital. During the year ten nurses had left the institution, seven of them to continue nursing elsewhere. Five of the six nurses working on the district staff of the Nursing Society had completed their term of two years, and had been replaced by others.

### The Nursing of the Insane.

BY A MEDICAL OFFICER.

THE number of nurses in our English asylums is roughly about 3,300. This estimate is certainly below rather than above the mark. The great majority of these are drawn from the class of domestic servants. The wages range from £16 to £25 annually, with board, uniform, &c. The scale of wages, and the amount of leave, &c., vary in different asylums. Up to a certain point, the present asylum nurse discharges her duties admirably. The phlegmatic, good-tempered girl from an agricultural district perhaps makes the best type of nurse. The nervous, impulsive woman is not suited to take charge of the insane. The calling would neither be good for her nor for those under her. Petty annoyances and insults which fall unheeded on the placid nature of the one girl, would goad the other to deeds or words which could not be overlooked. Were the girls from the country districts trained and educated for the work no better women for the purpose need be looked for. Unfortunately, the education is wanting before they are engaged, and the training too much neglected afterwards. The prospects before the present staff are a charge nurse's position and a pension. They, however, still look upon their work much in the light of domestic service, and are willing to leave it if tempted. The highest female posts are necessarily to a great extent closed to them, and from want of training and a true knowledge of their position, ambition, and enthusiasm in their work, do not influence them.

One respect in which mental nursing differs from the ordinary hospital nursing is that as a rule the nurse must select the asylum she is going to work in and must stick to it. It is only after being some time in charge of her ward that the nurse becomes most useful. She has then learned the peculiarities, whims, and cranks of her flock; she is accustomed to them and they to her and she can manage them with much less friction than it would be possible for a stranger to do. Superintendents are very chary of engaging a nurse who has served in another asylum, and as a rule, only do so on special recommendations. There is considerable force in the reasons they assign for this. The methods of one asylum are often essentially different from those of another, and each Superintendent naturally thinks his own is the best, and prefers those who have been trained up in it. As a rule the nurse who flits about is not a desirable acquisition. She generally has an unsettling effect upon the staff of the asylum in which she is temporarily staying, always contrasts her present situation unfavourably with her last, and, when she goes, leaves a feeling of discontent and dissatisfaction among the weaker members of the staff.

The wages given are too low considering the nature of the work and the responsibility incurred. The scale has been fixed more from a consideration of the class of girls of which the majority is composed than of the nature of the duties they have to undertake. £25 yearly may be at present considered good wages for an ordinary charge nurse to receive. When it is considered that she may have six or seven nurses under her, may have the care of 80 to 100 patients, and be held responsible for the stock of a ward for that number, it must be felt that the remuneration is not adequate.

Speaking generally the way in which the present nursing staff discharges its duties is worthy of all praise. The absence of cruelty and ill-treatment in our asylums is astonishing considering the provocation received. Like R. W.'s wife the lunatic is often very wearing. Yet if an accident happens how is it looked upon by the outside world? Nothing is heard of the trials undergone and the insults and violence borne, though often sufficient to qualify the tried one for a martyr's crown. No, as Dick Phenyl says, "Always blame, blame, but praise—Oh dear no." It must be granted that accidents occur occasionally. We are all mortal and are all frail. She who would successfully undertake the care of the insane must havh a patience unwearable. When reviled she must answer not again; when cursed must bless; when struck and spat upon suddenly and unexpectedly must have self control enough to subdue the unconscious impulse to resent and retaliate, and, hardest of all, she must have a mind strong enough to sustain her under false accusations and slanders. Open abuse and assault from a violent and excited patient is easily borne, but this misrepresentation by those apparently sane enough to deceive strangers is hard to bear. To know that after months of kind treatment and hard work you are, in the opinion of others, unkind, harsh, wanting in devotion to your duties and neglectful of those under your charge is indeed an occasion for the exercise of Christian virtues. If anyone prides himself or herself on his or her self control let them have it tried by a lunatic suddenly and without any warning walking up to them and deliberately spitting in their face. This is not an unusual experience for a mental nurse in our asylums.

More attention is being paid to the training of those in charge of the insane than formerly, but the movement is still in its infancy. The nurses have not yet fully grasped the idea that their calling is a different one from that of the ordinary domestic servant, and they have not yet begun to claim their rights. A lurking fear perhaps exists in the minds of some Superintendents that if their nurses were really trained, the best of them might take up private nursing, and so be lost to the institution. This is a selfish, an unscientific, and at the same time a short-sighted view. The Medico-Psychological Association have made an effort to introduce training of nurses and attendants, and after examination grant a certificate of proficiency. The nursing staff, however, evidently were not consulted in drawing up the plan, as they are entirely at the mercy of their Superintendents or the association. The nurse is still a bond servant, but at present theirs is the only scheme in the field, and the nurses are too scattered and out of touch with each other to do much in the way of organization. They require education and training: they want their eyes opened to the fact that while public sympathy has been expended lavishly upon their elder sisters in the hospitals they, though fully deserving as much consideration, have been passed by and the passers by have kept very much on the other side of the way. Even the advantages of the Nurses' Pension Fund are not generally known amongst them.

These stirrings among the dry bones will only be brought about by the influence of training, and by the admixture of more cultivated minds. The result in the end will be good, but for a while the minds of timid and conservative Superintendents will be perturbed.

### Appointments.

REDRUTH MINERS' HOSPITAL.—Miss Edith Fry has been appointed Matron to this hospital, the post having been rendered vacant by the death Miss Angove. Miss Fry, trained at the London Hospital and worked at Stratford-on-Avon and Aylesbury, then rose to be Night Superintendent at Derby, and Senior Sister at Devonport. Lately "Sister Edith" has had charge of the Dartmouth Cottage Hospital.

ALICE MEMORIAL HOSPITAL, HONG KONG.—Mrs. J. M. Stevens, who was trained in the Royal Infirmary, Edinburgh, and who holds the midwifery diploma of the Rotunda Hospital, Dublin, has taken up her duties at this hospital, to which she was appointed on the resignation of Mrs. Kwan Ah Mui, who had held the post from the opening of the hospital to the universal satisfaction of everybody. Mrs. Wong was appointed nurse-assistant at the same institution.

### Death in our Ranks.

AT Ventnor, on February 14th, 1892, Ellen Wright, aged 47. Nurse E. Wright, trained at the Royal Free Hospital. She then joined the Workhouse Nursing Association, and was appointed Night Superintendent at the Hampstead Infirmary, where she remained for 10 years. She died from heart disease and dropsy after influenza. Much regretted.

### Lady Doctors in India.

THE late Lord Shaftesbury used to say that London would be uninhabitable were it not for the charitable, benevolent, and Christian work carried on in it; and to imagine what this country would be like if there were no hospitals, dispensaries, nurses for the poor, and other agencies devoted to the alleviation of human misery and suffering, is calculated to arouse the most indifferent.

If medical mission work generally then is so important an agency, what shall we say as to its value in a land like India, among 139 millions of women, inconceivable numbers of whom being shut up in the zenanas, where social customs forbid any but women to enter?

The urgent need for such a work as this can readily be understood when the exceptional conditions of the women during sickness are considered. Their sufferings, extreme before, are considerably aggravated by the ignorant treatment of the so-called native women doctors, who are sent to their aid in order to keep off the evil spirits who are supposed to be tormenting them. Here, then, is a field well-nigh untouched and practically unlimited.

One of the oldest women's missionary societies in the world, founded in 1852, under the name of The Indian Female Normal School and Instruction Society, but now more generally known by its newer title, "Zenana Bible and Medical Mission," stands in the forefront of women's medical mission societies. It has upon its staff a larger number of fully-qualified lady medical missionaries holding British diplomas than any missionary society in existence, and commenced medical mission work so long ago as 1875, when a small dispensary was opened in Lucknow. In those days Indian women were not so accessible to lady missionaries as they are now, and it was not till 1883 that premises were secured for the purpose of a hospital with accommodation for in-patients.

It was about this time that the lady medical missionary in charge was called to render aid to the Maharani of Punnah, and the story is very well known—how the Maharani, having been successfully brought through a serious illness, sent a message to Queen Victoria, describing the sufferings of native women in times of sickness, and Her Majesty's answer to the missionary is also well known: "We had no idea things were as bad as that. 'Something must be done for these poor creatures.'"

Passing on from those days to the present time, the society has now three medical mission stations at Benares, Lucknow, and Patna respectively; at the two former places large and commodious buildings have been erected within the past few years, at a cost of some £6,000 to £7,000, the money being specially subscribed for the purpose; the Victoria Hospital for Women at Benares, a gift from one of the society's subscribers, and the Lady Kinnaird Memorial Hospital at Lucknow, in memory of the foundress of the Society. The medical mission at Patna is comparatively young, but the lady doctor in charge, together with an English lady nurse, find themselves overwhelmed with applicants for medical relief, and steps are being taken to erect a third hospital here. Nearly £2,000 has already been subscribed for this purpose, but another £1,000 is very urgently needed before the buildings can be completed.

The lady doctors undertake the most difficult surgical operations; thus in 1890, at Lucknow, 39 major operations were performed with very satisfactory results, death supervening in only two cases.

The lady doctors are assisted by an English Matron, native Christian trained nurses, and other Christian attendants.

There are also five ladies now studying in London, whose medical course is rapidly approaching completion, when they will at once proceed to the Indian mission field.

During the year 1890, the last for which particulars are to hand, the statistics for the hospitals at Benares and Lucknow are as follows: In-patients, 326; out-patients, 6,963; total attendances, 22,056; patients attended at home, 340; lady doctors' visits, 1,616. At Lucknow alone, for the past nine years, the in-patients have numbered 1,140; out-patients, 24,168; attendances, 68,000.

This work is capable of almost unlimited extension, and its value can scarcely be overrated. It is a work which provides for the relief of a mass of human suffering and misery, which few can adequately realise, and a work which has for its ultimate aim the salvation of the women of India. The office of the society is at 2, Adelphi Terrace, Strand, W.C., where help will be gladly received, or further information given.

### DAY BY DAY.

How few of us are satisfied with the events and circumstances of the present moment, instead of enjoying or making the best of them; we are always longing for the future which is going to be so delightful. Impatient to throw away the ills we know of, we are prepared to revel in anything we know not. And yet if we had learnt from past experience we ought to have realised that it is "distance lends enchantment to the view," for have we not generally found that what has looked so desirable afar is but a poor thing when approached closely. It is the old story, "Man never is but always to be blessed.

In illness we are still more susceptible to this impatience of the present, and indeed, very pardonably so. The flesh abhors suffering, and only the grace of God, renewed day by day, and not the unaided human heart will make us bear our trials cheerfully. Which of us has not seen patients with a broken limb who have bravely borne the torments of the "first aid to the wounded" become, as they near recovery, chafed and irritable because they must keep quiet to perfect the cure. "If I could only write or do something of the sort," says the proprietor of the broken right arm, but "I tire of reading, and am not strong enough to walk much; when I can get about again how I shall enjoy using my limbs." "You are better off than I am," says the owner of the broken leg, "I can hardly move without help, and my taste has never been for reading or writing." Both these invalids forget that circumstances alter cases, and that when they return to the active battle of life they will probably have too much of what they now desire, and will soon be sighing over the remembrance of the happy time when they had nothing to do but rest. Ah, yes, we carry ourselves about wherever we go, and it is alike our wisdom and our happiness to be contented with the present. It would not be good for us to have everything our own way. What poor weak creatures we should be if there were no difficulties to encounter; we should lose half the beauties of nature if we never climbed the hills. We must ask God to give us "day by day" our daily bread, and if we do it heartily He will daily feed both souls and bodies, and give us strength and help to bear whatever befals us. As thy days so shall thy strength be, is a promise full of mercy and of hope. We cannot have trials and pleasures at the same time, our sufferings and worries add by contrast to the enjoyment of the latter. So we may well carry out our daily round, our common task with cheerfulness. Come ye disconsolate to the mercy seat, for earth hath no sorrow that heaven cannot heal.

"Day by day" the promise reads,
Daily strength for daily needs;
Cast foreboding fear away,
Take the manna of to-day.

### A Private Asylum.

Some time ago in these pages appeared an article describing the Holloway Sanatorium at Virginia Water from the medical point of view; we propose now to look at it from the nurse's point of view.

The asylum itself stands close to the railway line, and is surrounded with a high brick wall; the grounds are extensive; there are several small airing courts for acute cases, other patients have the run of the whole grounds, others, even, are allowed beyond the grounds on their parole. Dr. Rees Phillips, who is the Medical Superintendent, receives visitors, and is responsible for the whole management. On the ladies' side, with which we are now chiefly concerned, are two medical assistants; the second of whom is Dr. Jane Henderson, late of the London School of Medicine for Women. It is an unusual, if not a unique thing for a lady doctor to hold the post which Miss Henderson has successfully filled for fifteen months, and it shows how advanced is the management at St. Anne's Heath, and how carefully considered is the comfort of the patients. The head nurse is Miss Builder, who has had twelve years of asylum experience, and holds the certificate of the Medico-Psychological Society; she has under her a female staff of about 50 nurses and housemaids, 20 of whom are lady nurses, chosen for their capabilities as companions, or, in the case of four, for their experience of hospital nursing. These lady nurses wear a grey uniform, with white cap and apron. Most of them can sing and play, one is specially employed in teaching her patients basket-work, others in teaching other sorts of fancy work and generally in amusing and occupying the patients and in keeping them bright and cheerful. The regular nurses wear a neat blue uniform. Lectures to the nurses are given by one of the medical men and by Miss Henderson. Last year five nurses went up for the medico-psychological, and all passed; this year another five are going up. The wards resemble long passages with numerous private sitting-rooms off them, and here and there a bay window, with a table and chairs, where a group of talkers or workers can gather. They are lit by electric light, the floors are polished, and the furniture and decorations are of the most comfortable and lavish description. All the appliances are wonderfully good; wire mattresses for the private bed-rooms, and Howe's beds, which are light to move, for the dormitories. The ventilation also was excellent; even in the acute wards was no trace of that peculiar smell of the insane we had come to believe was unavoidable. There is a large recreation hall where games of all sorts and books are provided, there is a billiard-room to which certain of the lady-patients have admission between tea and dinner. There are two dining halls, and in the evening the convalescent patients of both sexes dine with the doctors and the head nurse. We saw some of the sheets of daily returns from each ward which go to the head nurse, to be by her collected and formulated and handed on to the Medical Superintendent. They state not only how the patients have been so far as health is concerned, but how the day has been spent; thus, this person and that person have been out driving; so and so has received visitors in the garden, and so on. The system of nursing struck us as being very well thought out and carried out, there was no hurry or worry observable anywhere, and all the patients seemed occupied and happy. It is certainly the most comfortable asylum we have ever been over.

### Everybody's Opinion.

QUEEN VICTORIA'S JUBILEE INSTITUTE.

Miss Peter writes:—Under "District Nursing," in Hospital (March 19th) "Nursing Mirror," is mentioned that at Fort William meetings have been held in favour of establishing Queen's Nurses. Since October, 1891, a Queen's Nurse has been working in Fort William and neighbourhood with great success. Since January 1st, 1892, Queen's Nurses have began work at Annan, Berwick-on-Tweed, Dumfries, Dysart, and Milngaire, affiliating with Q V.J.N.I. Scottish branch.

### Examination Questions.

The worst set of answers we have ever received came in reply to the last question set, showing conclusively that sick-room cookery does not receive its due place in the training of English nurses. One nurse made her beef-tea of veal and mutton, and her name was not Eileen O'Brien; another nurse made her egg-flip with two pints of ale, and yet another of the whites of two eggs and a little lemon juice! A nice nutritive recipe that last one! We have awarded the prize to Nurse Josephine Taylor, of Caldicote Convalescent Home, Bushey, though her answer is not so full as we should have liked. For instance, egg-flip can be made, and usually is made in hospitals, with milk instead of water; and sherry or whisky can replace the brandy. Good answers were received from Nurse Edith Webb, Nurse C. G. Heath, Nurse Annie Reddock, Nurse Louise Frances, Nurse Douglas, and Nurse A. E. B. Williams. The prize for industry has been awarded to Nurse Esther Payne, of Coldstream Cottage Hospital, whose extremely neat and correct papers it is a pleasure to read. We give the prize answer below.

Examination Question for February.—Prize Answer.

Beef-tea.—Ingredients: One pound of best gravy beef, one pint of cold water. Cut the beef into moderately small pieces, taking away all fat; put in a jar with the pint of cold water, cover it, and let it stand for an hour; then place the jar in a saucepan of boiling water, which must be kept filled up and boiling for four or five hours. Then strain through a moderately coarse sieve, so that only the lumps of meat are kept back, and not the nutritive brown particles. If wanted at once, the fat may be taken off by strips of clean tissue paper over the surface; otherwise it can be skimmed off when cold.

Egg Flip.—Ingredients: The yolks of two eggs, one ounce of brandy, a little white sifted sugar, flavouring. Beat the yolks up with the sugar into a light froth, then add the brandy; a little cinnamon and water mixed may be added as a flavouring.

Peptonised Gruel.—Ingredients: Two tablespoonfuls of prepared groats, one pint of milk, two teaspoonfuls of Benger's liquor pancreaticus, half a level teaspoonful of bicarbonate of soda. Make a good gruel of two tablespoonfuls of groats to a pint of water, add to it a pint of cold milk; to half of this mixture, *i.e.*, one pint, put two teaspoonfuls of the pancreaticus and the bicarbonate of soda, and let it stand for the process of digestion for from ten to twenty minutes according to the nature of the patient; it will be then ready for use, and the digesting process will continue when in the stomach. If all is not required to be taken at once, the remainder must be brought at once to boiling point, for at that temperature all digestive action ceases. A little coffee hides the somewhat bitter taste.

The following examination paper for the nursing certificate of the Medico-Psychological Association given in November, 1891, will be of interest to our readers in asylums:—

*Digestion.*—Describe briefly the process of digestion.

*Nervous System.*—Name the chief parts of the nervous system. State where they are to be found in the body. Give a short account of their composition.

*Temperature.*—How would you take the temperature of a patient? State in figures what variations in temperature you would specially report to the medical officer.

*Fomentations.*—What are fomentations? How are they applied? What precautions should be observed?

*Wet-Pack.*—Describe briefly the process of packing in the wet sheet.

*Hanging.*—What is the first thing to be done on discovering a person hanging by the neck? What is the method of treatment if the breathing has ceased?

*Insanity.*—What do you mean when you state that a person is insane? How do you judge of a person's mental state?

*Epilepsy.*—What are the chief features of epileptic insanity?

*Delusions.*—How should an attendant behave in regard to the delusions of patients?

*Struggles with Patients.*—Why is it best to avoid a single-handed struggle with a patient?

*Special Reports.*—Enumerate the principal emergencies in which you would make an immediate or special report on the state of a patient.

*Intellectual Faculties.*—What do you understand by the intellectual faculties?

### "Mrs. Pharisee."

I ALWAYS called her that in my own mind, and the name fitted her beautifully. She was a patient of mine while I was training at the Maternity Hospital, and would have afforded intense amusement had not the grim realities I was brought face to face with for the first time almost quenched my sense of humour.

A good, honest, respectable woman, and a devoted wife and mother, she belonged to that intensely narrow type of woman who have no pity or tenderness for the weaker ones, and it was impossible to help associating her with the "Pharisee" in that wonderful old story.

She was quite a character in her way, and certainly "cumbered with many cares." I have often smiled since over the first letter I wrote at her dictation. As far as I can remember, this was it:—

"Dear Husban',—Me an' Baby are doin' nicely, but I was very bad. She is a girl, an' nurse says her eyes are like a kitten's. Tell Tom to mind not leave the soap in the water, and make Annie comb her hair. I'm dreadful sorry I could not stay to mend your socks—the yeller ones has less holes in them than the brown. Mary is to give the district lady back the trac' under the tea-caddy when she comes, and ask for 'Eternal Fires.' There's some treacle in the blue chiney jug on the top shelf the children can have for tea. Mary has the button off your Sunday coat. . . ."

My other patient—we had, as a rule only two in each ward—was of quite a different stamp—"a woman with a history," and yet with a face that was gentle and refined, and eyes that looked as if they knew no evil. Poor Lizzie. She was such a gentle creature, and so pathetically grateful always. I remember how once, when I was doing her some small service, she looked up at me with a perfect passion of gratitude in her thin, white face. "Oh!" she said, under her breath, "and you don't mind doing it for such as me? . . . '

Knowing her story, one would have thought she had been thankful that her child was dead. But the instinct of "mother love," so strong in some women's hearts, had stirred within her, and, with the little dead baby, she seemed to have lost her one hope of comfort. Poor thing—does anyone know, I wonder, what life means to such as these?

One could not help being fond of her. I often longed to shake Mrs. Pharisee, who took everything as a matter of course, and sniffed at poor Lizzie in the loftiest way. I did come down upon her once. Lizzie had asked some timid question, and Mrs. Pharisee had ostentatiously turned her back and refused to answer. I waited until Lizzie was asleep, and then I relieved my mind most vigorously. It was touch-and-go whether Mrs. Pharisee would resent or own the justice of my rebuke, but her conscience evidently accused her, for she muttered something about "not meaning it" and "speaking kind-like" in the morning. But her penitence was not deeply rooted, and, though she was never openly rude again, she yet managed to convey her disapproval in the most emphatic manner.

The last evening came, and Mrs. Pharisee undid the small bundle of clothes she had brought for the baby to leave the hospital in. Such threadbare little garments they were, neatly patched and mended, but almost falling to pieces from hard usage, and she looked at them ruefully as I hung them in front of the fire to air.

Lizzie had asked for her bundle, too, and now the things were lying on the coverlet. So tenderly she touched them with her wasted fingers—the little blue head flannel, daintily worked with silk, the tiny shirt and night gown tucked and feather-stitched as if they had been made for some happy woman's baby, and the wee socks with their smart white ribbon bows. "I would have loved it so," she whispered to herself, a great tear falling upon the little night-gown.

The clock struck seven—it was time to settle both my patients for the night. A timid tear-laden voice came from Lizzie's bed. "Nurse," it said, with a little sob between the words, "I want—the other baby—to have my things. Please ask its mother to let it! . . . ." I had never liked Mrs. Pharisee until that moment. She threw one glance at the little heap of clothes she had been eyeing so enviously, and then she literally "lifted up her voice and wept." Of course Lizzie cried too, and I'm afraid I was nearly as bad myself. It was so pathetic—and I am quite sure that out of us three it was Lizzie then who was nearest the "Kingdom of Heaven."

Some months later I was hurrying across Wimpole Street, very much intent upon my own "troubles and cares," when I heard a rapturous cry of "Nurse!" and someone dropped a big parcel in the middle of the road. It was Lizzie, looking brighter and happier than I had ever hoped to see her. The old sorrow still shadowed the sad grey eyes, but her face was no longer drawn and haggard, and she looked as if life had begun to be worth living to her again. She was "in service," she told me shyly, with some very kind people, and spent her Sundays out "with—Mrs. Pharisee!"

### Where to Go.

SATURDAY was Show Saturday, and we wended our way, where report told us we should have a treat, to Miss Maud Earl's Studio, in Bloomfield Place. Our expectation was more than realized. Miss Earl's pictures are really masterly. She devotes her attention principally to the portrayal of dogs, and that her studies are *con amore* is very evident. Above all, has she learnt the varying moods of the beautiful fox-hound, and many are the interesting stories she can tell us of their behaviour as occupants of the studio. Of the three principal pictures we had the pleasure of viewing, "Ware Riot" took our fancy most. The eager, rushing pack are literally bounding in air, as they leave the kennel doors with eyes brilliant with excitement and expectation, and the technique is as excellent as the conception of the picture. It will be strange, indeed, and a loss as well, if we do not see this picture on the walls of the Academy. "Who Comes Here?" is a study in expression, and "The Tocsin of His Soul—The Dinner Bell," is a most clever study. Miss Earl shares her studio with Miss Smythe, whose excellent etchings are familiar to nurses of the Royal National Pension Fund, who each possess a specimen in their certificate.

### Notes and Queries.

### Queries.

*Home Wanted.*—Would any home receive a young woman of twenty subject to epileptic fits; able to pay 20s. to 30s. weekly.—*Sister, Nurses' Home, Plaistow, E.*

*To Oxford Readers.*—Is there any place in Oxford where massage is taught and a certificate given?—*Grannie.*

### Answers.

*Mrs. K. C.*—You must tell us for what nursing institution or parish you work, if you wish us to insert your appeal.

*Matron.*—Apply to St. John's Home, Cowleigh, near Malvern. For list of convalescent homes, and full particulars, see "The Hospital Annual." Bromsgrove Cottage Hospital might take the case for a small payment.

*S. Janet B.*—The training is good; you would probably find it difficult to get into a hospital afterwards, but there are excellent posts to be had in the infirmary world—such as that of Matron to the Birmingham New Infirmary for instance. You write a good letter which would be in your favour.

*Paraffin Stain.*—If the carpet is a woollen one Chiver's carpet soap will remove the stain.

*Sister Dora.*—You had better go to Debenham and Freebody, of Wigmore Street; they are used to Indian nursing outfits. Probably £15 would cover it.

*Asylum Attendants* communicating with the Medico-Psychological Association respecting certificates, should address to the Hon. Secretary, Mr. F. Beach, Darenth Asylum, Dartford. Letters have been received addressed to Hanwell, and delay has consequently occurred.

*Mass.*—A. Creighton Hale's book not out yet; will be ready shortly. We believe the price will be 6s. Male nurses are trained by the Hamilton Association, 57, Park Street, Grosvenor Square.

### Magic in Medicine.

To the great majority of our forefathers, there was something far more fascinating in a cure effected by the operation of the miraculous than in one brought about by the application of science; hence it is that we find magic nominally made use of in the practice of medicine at a period when belief in the utility of the black-art was fast dying out, or was already dead. We say *nominally* made use of, because it seems probable that some of the men who professed the practice of magic in medicine, did so simply for the sake of their pockets; and in reality made use—though unacknowledged use—of the resources of science in the treatment of their patients.

It was to put down this scandal and to give honest men a chance of making a livelihood, that the College of Physicians was established, with powers to proceed against and punish empirics of every description. The College once established, the poor, though learned, practitioner who, as we suggested, to keep a practice together, hinted to his patients that alchemy was numbered amongst his accomplishments, felt that his unlearned rivals would ere long be suppressed, and therefore did not continue to claim acquaintance with magic. That fact—together with the fear of prosecution—soon caused him to acknowledge no other agency in effecting his cures than that of medical science.

But the putting down of his unlearned rivals—"quacks" pure and simple—took the legal arm of the College a good deal longer time than was expected. Founded in 1518, we find it, as late as the close of the seventeenth century, pursuing its crusade against the successful "quack," and—what is more—pursuing it under difficulties thrown in its way by those in "high places." In 1585 the College complained that the two universities granted medical degrees to persons who did not come up to the proper standard of efficiency. Elizabeth and her Ministers often interfered in the punishment of those condemned by the College. When Charles II. "directed" the College to desist from the prosecution of a Dutch quack—Geard Van Mulen by name—the answer made to the merry monarch was that he had better mind his own business—put, of course, into polite language!

Very quaint are the records of some of these proceedings against "Quacks" as one reads them in the College annals. In 1571, during Caius's presidency, the wife of one Skeres was sent to prison for treating bad faces with a saline wash, which, said her patient, had "spoiled her face." Though useless, the compound did not appear to the College harmful, and Mrs. Skeres got off with no worse punishment than having to pay ten shillings damages to the lady who lost her complexion. The same year a Spanish doctor had to return the money he got from one of Burghley's servants for fraudulently undertaking to cure his swollen shin-bone. Soon after, Roger Powell, an advertising doctor, got into trouble, and we find him cited to answer for fixing "bragging bills" on the walls of houses.

In March, 1593, Simon Foremann, "of the county of Wilts," appears on the scenes; he admits that he has practised medicine in England for sixteen years, and in London for two years. He boasted that "he used no other help to know diseases than the Ephemerides, and by celestial signs and aspects and constellations of the planets, he can at once understand any disease." By direction of the College he was therefore examined in astronomy, and found therein "laughably ignorant." He was interdicted from practice, and fined £5, which he promised to pay within sixteen days. Two years later Simon was again before the College Council for continuing to practice. This time he was examined in medicine. He knew as little of that as he did of astronomy. He was sent to prison and fined £10, but—here is a clear case of interference by a "friend at Court"—was released by authority of the Keeper of the Great Seal!

Another prosecution followed next year. Simon this time confessed "administering a water composed by himself to a Mr. Sotherton in a burning fever, and that he immediately died." Again he pleaded that he knew diseases only by astrology. His impostures were now too palpable. He was sentenced, and his imprisonment was not meddled with. For how long he was imprisoned we do not learn, but he was at large and at his old tricks in 1607. He was then again cited, and on this occasion his "treatment" is minutely described: "First he asks the name and address of his consulter. Secondly, he erects a figure. Thirdly, like a prophet, he judges of the disease and the event. Lastly, he prescribes a medicine." Though cited, Foremann would not come, and this is the last we hear of him save as to his charges, which, it need scarcely be said, were exorbitant to a degree, though not quite so exorbitant as those of another quack, who, about this period, charged £6 for a pill!

"Touching" for the king's evil yielded a rich harvest to the "quacks." Here are a few facts regarding one Leverett, whom the College prosecuted in the Court of Star Chamber. He tells his own story. He is, he says, the seventh of eight sons, "but doth not challenge any virtue by being the seventh son," a curious testimony this to the antiquity and belief in the healing powers of a seventh son, still prevalent amongst the miner folk in Cornwall. In practice he used these words: "God give a blessing; I touch, God heals." He asserted that after touching thirty or forty persons in a day, "he finds himself weakened, by the virtue which goes out of him, more than when he was a gardener by digging up eight roods of ground." He could not "touch" when his hands were cold. He was called upon to give a public demonstration of his cures, of course failed, and the College pronounced him an impostor, whose "pretended cures and the manner of them" were "full of superstition and sorcery."

Space forbids us quoting later instances of proceedings against quacks by the College of Physicians; but they are to be found, and, as we have already said, those "in high places" too frequently interfered in carrying on the good work. We all remember with what favour good Queen Anne regarded the famous quack eye doctor, William Read, who, possessed of a great deal of "cheek" and very little knowledge, managed to get together a comfortable fortune, and received knighthood. As with the sovereign, so with the aristocracy of the time. It is a mistake, quite a mistake, to imagine that the poor or the uneducated alone supported the quacks. In consulting them—if they did so—they were following the example of their "betters." Lord Strafford, one of England's ablest diplomatists at the outset of the last century, who steered his country through heaps of difficulties and dangers, could not in after years steer himself clear of a country quack who was always hanging about his Yorkshire seat. "I know," writes one of Strafford's intimate friends in urging him to consult a London physician, "you hate physick and physitians, and love quacks, but I shou'd think that you had suffered enough by them to grow wiser for the future. . . . For God's sake resolve never to meddle with quacks again as long as you live." But, like most good advice, this was not taken.

## Figures and Tables

**Figure f1:**